# Communicating the Risks of Air Pollution to the Public: A Perspective from Jordan and Lebanon

**DOI:** 10.1177/11786302221127851

**Published:** 2022-10-18

**Authors:** Rami Saadeh, Yousef Khader, Mazen Malkawi, Mohammed Z Allouh

**Affiliations:** 1 Department of Public Health and Community Medicine, Faculty of Medicine, Jordan University of Science and Technology, Irbid, Jordan; 2 World Health Organization, Regional Office for the Eastern Mediterranean, Centre for Environmental Health Action, Amman, Jordan; 3 Department of Anatomy, College of Medicine and Health Sciences, United Arab Emirates University, Al Ain, UAE

**Keywords:** Air pollution, communication, health, awareness, interview

## Abstract

**Background:**

Communicating air pollution to the public is essential in reducing exposure to air pollutants through increasing awareness and promoting precautionary actions. However, one way to approach the public is through healthcare professionals who are considered public health leaders and could influence the public’s opinion. The current study aimed to investigate the perception of health experts about communicating air pollution to the public.

**Methods:**

Personal interviews of 32 health professionals were conducted to report their opinions about communication of air pollution through an open-ended questionnaire. Interview questions were focused on 5 themes: common air pollutants and health risks, goals and barriers of communication, types of information to disseminate, target groups, and vehicles of communication.

**Results:**

Interviewees agreed that air pollution should be communicated to the public. Major barriers to achieving effective communication were people’s poor comprehension and lack of interest of policymakers. The levels of pollution, associated health risks, and ways to protect one’s self were the most frequently reported types of information to distribute. Most interviewees focused on patients with pre-existing conditions and children as the main target groups. Further, social media and text messages were preferred as vehicles of communication.

**Conclusion:**

Although not all interviewees had a clear idea of how to develop and implement a communication system, most of them agreed on its importance in protecting the public. More emphasis on this topic and further investigations are expected to increase the interest of health care professionals in communicating the risks of air pollution and advocating for public health policies regarding air pollution.

## Introduction

Air pollution is causing significant adverse effects on health globally. The World Health Organization (WHO) reported several health conditions associated with increased levels of air pollutants.^1^ Air pollution is becoming a global issue, and both developed and developing countries are suffering from the health, economic, and social consequences resulting from increasing levels of air pollution.^2^ The Arab region is not an exception. It exceeded the WHO recommended pollution levels, surpassing the recommended pollution limits by 5 to 10 times more,^3^ which is attributed to the lack of enforced standards on emissions, the increased demand for electricity, and the limited use of environmental-friendly resources.^4^ Some efforts have successfully reduced air pollution in the region, such as producing unleaded gasoline and decreasing the use of diesel fuel, resulting in lower sulfur content in the air. However, pollution is still exceeding the safety levels of many developed countries.^5,6^

To decrease the impact of air pollution on populations, leading organizations took action by preaching coping behaviors and recommending policies that protect the public from further exposures. The WHO, for example, had proposed that countries should have stricter air quality standards, implement structural changes (eg, planning of land use, clean energy, transportation modes), and inform individuals about protecting behaviors to avoid unnecessary exposures.^7^ The disseminated information included air pollution weather forecasts, predicted or observed exceedances of alert thresholds, groups that are sensitive to air pollution, related health risks, recommended health behaviors, precautionary actions to reduce exposure, and encouraging advocacy for clean air through changing policies.

Unfortunately, a system of disseminating information about air pollution is lacking in the Arab region, and efforts in that direction are scattered and limited. Although it’s necessary to launch a system that disseminates information to the public in the region, challenges encountering this process are unknown, mainly because no system was ever established. Some of the challenges seen in the European countries included the generation of good quality air pollution data, the low level of public awareness about air pollution, the ability to use services related to air quality information, the high variability of the public’s perception about air pollution, lack of proper training for health professionals required for effective communication, and poor assumptions about target populations.^8-10^ In addition, there are vulnerable populations at a higher risk of air pollution, which should be carefully addressed in the communication. Higher dissemination of information about air pollution among the public and vulnerable increases their awareness and improves their risk perception.^11^ Hence, it’s necessary to start studying factors that could produce an effective communication system in the region, because the challenges of proper communication, like political well and people’s cooperation, could hinder the process of communication and reduce its effectiveness. One approach to advocate for cleaner air and communicate with policymakers, as well as the public, is through healthcare professionals and air pollution experts, who are considered public health leaders, and could have an influence on the public’s opinion. Thus, this study aimed to investigate the perception of health experts about communicating air pollution to the public, which shall assist in identifying critical key elements needed to establish an effective communication system for air pollution.^12^

## Materials and Methods

### Design and settings

A qualitative research design was the adapted approach for the current study. The items of the research questions (Appendix 1) were developed to answer questions related to the opinions of health experts on topics related to communicating air pollution. The subjective views of the interviewees were gathered in a narrative format and organized to form themes and sub-themes that represent the interview guide (Appendix 1).

A total of 33 (23 from Jordan and 10 from Lebanon) air pollution experts and physicians who specialized in treating diseases associated with air pollution were invited to participate in this study. One person from Jordan refused to participate in the study, and semi-structured interviews were conducted with 32 experts. We selected our interviewees after reviewing their work in the area and their expertise in diseases related to air pollution using the snowballing approach. Snowballing technique is a non-probability sampling technique that relies on referrals from other participants in the study who share similar research interests and experience in the field.

Both countries (Jordan and Lebanon) fall within the same WHO administrative region. They share many cultural and ethnical backgrounds, yet there is a quite difference in the level of air pollution; Lebanon reports higher air pollution and lower air quality index.^13^ In addition, Lebanon faced difficult economic conditions that hindered the advancement of the country in many aspects, including improving air quality.

### Data collection

Interviews were done face to face in work offices and were conducted by the same researcher.

The purpose of the study was explained to participants before starting the interview, and interviewees were informed about recording the interview. The willingness to participate was obtained through oral consent. Interviews were completed between December 2018and January 2019. The duration of the interviews ranged from 8 to 23 minutes.

### Characteristics of interviewees

There were 32 participants interviewed in the study. They were 5 cardiologists, 5 pulmonologists, 4 allergy/immunology physicians, 1 pediatrician, 1 thoracic surgeon, 1 nursing professor, 1 nephrologist, 2 endocrinologists, 3 environmental health specialists, 3 epidemiologists, 3 family medicine physicians, 2 toxicologists, and 1 physics professor who has direct experience with air pollution research in Lebanon.

### Interview guide

The interview guide contained detailed questions developed from studies in the literature pertaining to air pollution perception and communication.^14-16^ Although study participants were proficient in English, the interview guide was translated to Arabic for their convenience. The Arabic translation was translated back to English to check the validity of the questions. The translated version (Arabic) was validated by 2 public health experts and tested with 1 participant to review the understanding and reaction to questions, time taken to complete the interviews, and concerns or comments.

There were 9 semi-structured questions that focused on 5 major themes: (1) common air pollutants and health risks, (2) goals and challenges of communication, (3) type of information public and patients should be aware of (4) vehicles for conveying information, (5) and intended audiences. Some of these themes were integrated into more than 1 question to examine an in-depth opinion about each of them within different contexts. All interviews were recorded after taking the consent of participants.

### Data analysis

A deductive thematic coding approach was used to analyze and categorize themes used in the interview guide. Codes were deducted from the responses of each participant that were transcribed and grouped into their corresponding question. These typed transcripts were used to code the responses and then grouped into themes.^17^ Codes were counted to describe the proportion of participants who agreed/disagreed or supported/didn’t support a certain aspect or concept within the theme and to explore the magnitude of the issue within each category. Comparisons were made for the following: percentages of those opposing a concept from the total number of participants, comparisons between the 2 countries, and comparisons between different areas of specialization. Key messages were utilized in the text to highlight important information. Those key messages were statements of participants illustrating a key message or supporting a specific theme. The selection of those statements was based on the level of information contained in the statement that represents the theme or sub-theme analyzed.

### Ethical considerations

The Regional Office for the Eastern Mediterranean, Centre for Environmental Health Action, Amman, Jordan, had approved this study. Oral informed consent was obtained from all participants. They received an explanation of the objectives of the study and were ensured that the obtained information was anonymized and confidential. In addition, they were informed that their participation was voluntary and that they could withdraw at any time.

## Results

### Health risks and sensitive populations

We questioned our respondents concerning their knowledge about the impact of air pollution on health and sensible populations. Most of the participants (94%) reported lung diseases, including asthma exacerbations, chronic obstructive pulmonary diseases (COPD), lung fibrosis, and interstitial lung disease as diseases associated with air pollution. Approximately, two-thirds believed that an association of asthma with air pollution exists (n = 20, 63%), and half of the participants reported allergies, including allergic rhinitis, skin allergy, atopy, and conjunctivitis. Lung cancer and other types of cancers were reported by 10 (31%) interviewees, and cardiovascular diseases by 8 (25%). Further, mental irritation, pregnancy outcomes, and endocrine disturbances were reported by 7 experts.

Regarding target populations (ie, groups expected to have a higher sensitivity to air pollution), children and individuals with pre-existing conditions were the main 2 groups reported (63% and 67% respectively), followed by elderly and pregnant women (29% and 13%, respectively).

### Main air pollutants and groups at higher risk of exposure

When participants were asked about the main air pollutants that could affect people’s health, few mentioned the criteria for air pollutants set by the United States Environmental Protection Agency^18^ (Table 1), and most of them considered smoke expelled from cars or factories as a common source of air pollution. Another main pollutant reported by many interviewees was dust. Other reported pollutants were tree pollens and emissions from electric generators or domestic waste incinerators, which is a common problem in Lebanon. Further, most interviewees considered people living near factories, highways, and crowded areas, to be more vulnerable to air pollution than others, in addition to drivers and those spending most of their time on the roads.

**Table 1.** An overview of responses for selected themes categorized by different specialties.

Specialty	Main health risks	Main air pollutants	Necessity of communication	Vehicles of communication	Challenges of communication
Cardiology (n = 5)	Respiratory diseases (75%)	Smoke, dust (75%)	Not very supportive, hopeless toward its benefits (100%)	Mainly social media (100%)	Public comprehension and response (75%)
Pulmonary (n = 5)	Respiratory diseases (100%)	Pollens (100%)	Supportive. Aim is to increase awareness (100%)	Social media and text messages (100%)	Cost of establishing a monitoring system and generating data. Public resistance (75%)
Immunology/allergy (n = 4)	Respiratory diseases (75%)	Different pollutants (100%)	Supportive. Aim is to increase awareness (100%)	Mainly social media (100%)	Establishing a monitoring system, and government interest (50%)
Public Health (n = 6)	A wide variety of disparities was mentioned (100%)	The six air pollutants criteria (33%)	Supportive. Aims are to increase awareness, reduce pollution and increase protection (100%)	Mainly social media (100%)	Public comprehension, costs, government interest, monitoring system, persistence of sources of pollution (50%)

This table summarizes the overall opinion of interviewees about themes discussed in the interviews.

Percentages in each cell represent the number of interviewees for each specialty who shared the opinion mentioned in the cell.

### Scope of communication: Goals, importance, and challenges

Most interviewees agreed on the importance of communicating the health risks of air pollution to the public. However, some experts were not comfortable with the idea of communicating air pollution with the public at this stage; claiming that communication will not change anything because of the lack of an established monitoring system and governmental interests in legislating and/or minimizing air pollution, which thereby, could cause panic among people; for something harmful that they can do nothing to stop it. In that sense, 1 of the interviewees said: *“I don’t know, not sure, because it depends if communication helps to reduce sources of pollution. There is a lack of communication between people and the government”*. Another participant contributed: “*I don’t think there is a benefit from communication if policymakers are not aware of these issues, and patients are not cooperative*.”

The goals of air pollution communication with the public were mainly to increase awareness of risks of air pollution (74%) or to reduce air pollution (67%). Other goals of communication, as reported by some interviewees, were to encourage the public to participate in the decision-making process for policies related to protecting the public from the harmful effects of air pollution.

Challenges of establishing effective communication with the public were numerous, ranging from the difficulty of building a monitoring system of air pollution to the point of having difficulty with the comprehension of the public about information related to air pollution, and cooperation or acceptance of such information. Almost all Lebanese interviewees stated that comprehension of the public is not considered a problem in Lebanon because the public is fully aware of the issue of air pollution. Instead, Lebanese interviewees reported the lack of political interest, difficulty in convincing policymakers, the cost of generating data, and the sensitivity of sharing data with the public to be the major possible challenges expected when establishing a communication system. Another important point that many Lebanese interviewees repeatedly mentioned was the difficulty in discarding electric generators, which are considered a main source of pollution in Lebanon. In Lebanon, the shortage of electric coverage is compensated by private electric generators, and it’s currently not an option for people or the government to forsake these electric generators, which produce high amounts of particulate matter. Thus, their continuous production of emissions hinders the possibility of providing clean air in Beirut, the capital of Lebanon. On the other hand, Jordanian interviewees focused on the lack of interest among the public and the low level of comprehension or cooperation for any recommendations provided to reduce the risk of air pollution. Moreover, some physicians expected resistance from the public toward changing their behavior based on their experience with patients who frequently overlook instructions provided by their physicians and lack interest in learning about their medical conditions.

### Vehicles of communication

There was a high level of agreement among participants that social media is the most common and influential source of communication nowadays (Table 1). The second most common vehicle of communication was text messages, which was preferred by some participants as a direct way to reach sensitive groups during dust storms and episodes of high air pollution; to re-emphasize instructions previously provided, including the use of allergy medications in advance and other medications related to diseases expected to get worse by these storms. Text messages can be text phone messages or WhatsApp messages, a common social media mobile application. Some clinicians reported using this application with patients on a routine basis to consult them with medications and answer their questions. One expert advised that a special phone number should be provided to sensitive groups or patients with pre-existing conditions to answer their inquiries. “*This service should guide them if they need help*,” he commented.

Television was another vehicle of communication preferred by few experts. They claimed that it is more effective for: older people, those who don’t have an internet connection, or those not interested in social media. Another way of communication was through campaigns, which had conflicting opinions among participants since some supported this method while others pointed out that their previous experience with this method was not successful. Moreover, few had mentioned the electronic street panels, which are electric signs that provide daily readings on air quality. These electric panels or signs were described as an effective and modern way of providing information about air pollution.

It’s noteworthy to mention that 2 interviewees indicated the necessity of investigating the best vehicle of communication before selecting 1 to use. They believed that designing studies that examine the response of the public to different ways of communication provides evidence-based facts about the most suitable and cost-effective vehicles of communication to be used.

In general, responses of interviewees showed that their preference in choosing the best vehicle of communication is based on their belief that the vehicle is widely used, readily available, provides daily access, and could reach the maximum number of people.

### Type of information

The type of information or the content of the message that should be transmitted to the public was explored in 3 different questions:

Information about air pollution, including the way levels of air pollution should be communicated, such as a scale, indices, percentages, etc., whether names of air pollutants should be mentioned or not, and any other information that the interviewee view as important.

The majority of experts agreed that messages should include levels of air pollution (74%), mainly represented as a basic ranking scale, such as low, moderate, high, or expressed as colors that change based on the pollution level. Only 3 interviewees reported that air pollution levels should be provided in concentrations or numbers, accompanied by normal limits for comparison. Some agreed that names of air pollutants should be provided, and others didn’t see a benefit of mentioning names unless they are explained within the context of their associated health risks. Providing sources of air pollution was considered necessary by some interviewees, so people can act to reduce them. Some also recommended sending messages on possible actions that the public can take to reduce air pollution, such as using green energy and advocating for clean air through communication with policymakers regarding air pollution.

Moreover, most experts agreed about the importance of relating air pollutants to their associated diseases or conditions when sending messages. Some clinicians recommended providing signs or symptoms of diseases exacerbated by air pollution so that patients can seek medical help. One of the interviewees mentioned that providing the name of the pollutant that reaches high levels accompanied by its known health risk(s) is the best way of communicating the health impacts of air pollution.

Actions for protecting oneself against the adverse effects of pollution were using masks during episodes of high air pollution, staying home, and seeking medical help if needed, during high episodes of air pollution. However, many emphasized the importance of reducing sources of pollution rather than advising people to avoid it—since the problem will not be solved by avoiding it. They commented that asking people to stay home while they should go to work will not be the right decision and that managing air pollution is the responsibility of the government.

Few had remarked that if the pollution is natural, such as dust storms, we can accept the idea of staying home because we don’t produce it. On the other hand, man-made pollution is the responsibility of the government, which should fix it rather than asking people to stay home. Another commented on the same topic, saying: “*there is not much that people can do. It will be worrying people on an issue that is important to which we have no response, and as a public health practitioner, I don’t like to create the awareness of a problem when no solution is proposed to it.”* Public health professionals, in general, were frustrated by the levels of air pollution. They criticized the idea of proposing the approach of communicating air pollution while finding ways to reduce pollution is generally not discussed.

### Information provided during dust storms or episodes of high pollution

Almost all interviewees recommended that patients with pre-existing conditions and sensitive populations should be advised to stay home or indoors during dust storms. Another comment made particularly by physicians was to remind patients about preparing or reinstalling their medications and use them in advance. Most interviewees, however, recommended the same message for the public, while some specified it for target groups only. One Jordanian cardiologist, for example, provided that she wouldn’t ask the public to stay home during dust storms but would do so to her patients. Moreover, 1 pulmonologist claimed that his patients already knew what to do during dust storms and didn’t see a need for special messages during these storms.

## Discussion

This study opened a great opportunity to receive a myriad of thoughts and opinions about a critical global health issue in the region. Discussing air pollution with all its components is a step forward to set this topic as a priority within the long list of public health issues that the region suffers from. The open-ended questions used in the interviews offered a wide variety of answers, which provided a platform for key issues about communicating air pollution, as well as some details that could not be obtained without this approach.^19^ In addition, the blended selection of interviewees added credit for viewing the issue from different perspectives.

The themes selected for investigation in this study were considered core points in communicating air pollution, as reported by several studies.^8,14,19^ Two main themes discussed in this study were goals and challenges of communication. The majority of responses related to the goals of communication were focused on increasing awareness and reducing air pollution through coping behaviors and advocacy, which were similarly reported by other studies.^14,19^ However, a study conducted by McLaren and Williams^20^ showed that air quality forecasting services provided to patients with respiratory diseases were not effective in reducing hospital admissions. Nonetheless, communication with the public is still believed to play a major role in reducing risks associated with air pollution. This is reflected by the efforts of the federal government in Australia that called for public consultation in the revision of the National Environmental Protection Measure (Ambient Air Quality) for particulates.^15^ Further, Kelly and Fussell,^21^ in a review study, stated that most studies on the topic concluded a positive impact of educating the public about the relationship between air pollution and related illnesses. However, fewer studies had concerns about the effectiveness of public involvement in reducing air pollution.^21^

Regarding communication challenges, the responses of interviewees focused on either public comprehension and coping behaviors, which were reported by some studies,^16,19,21^ or the ability to change policies, as reported by other studies.^7,14,19^ The difficulty in changing policies related to air pollution is expected in Jordan and Lebanon because of the low level of awareness about the topic and no interest among policymakers since new problems emerge frequently. In Lebanon, for example, wars encountered in the last few decades and the unstable economy made politicians less concerned about air pollution and other environmental problems that people are aware of, like the private electric generators, which produce a considerable amount of pollution, the garbage waste crisis, and the massive explosion at the Port of Beirut in 2020 where large amounts of ammonium nitrate were stored.^22,23^

Although developed countries had already launched a system or approach of communicating air pollution to the public, their follow-up studies reveal important points that were lacking or need improvement within the process of communication. It was concluded, by many studies, that further considerations must be included in the process of communicating air pollution to consolidate the efforts and enhance public involvement.^8,14,16,19,20,24-26^ One example is a study conducted in Spain, which assessed the process of communicating air pollution in Spain by looking into 2 aspects: (1) opinions of 20 experts and (2) the assessment of available approaches used in communicating air pollution in 4 Spanish cities. The authors of that study recommended the necessity of implementing evidence-based regulatory, structural, and behavioral interventions that incorporate findings from social and behavioral sciences to achieve holistic and effective protective interventions.^19^ Another study by Johnson^26^ found that despite the availability of the Air Quality Index (AQI) in Patterson, New Jersey, some people might not have access to it. More seriously, clear definitions of air pollution were lacking, in addition to poor explanations for sensory perception of the public, vulnerability, and health consequences.^26^ A comprehensive report released by the Pollution-Related Diseases Program of the European Commission, entitled “Air Pollution and Health: A European Information System” (APHEIS), concluded that to improve communication with the public about air pollution, there is a need to develop different communication tools (reports, brochures, slide presentations and so forth) in addition of including various types of content, then direct each of these tools and its content to target groups based on their needs.^9,27^ The findings of such studies shall be useful when developing a communication system for air pollution in the region while considering the cultural context, including people’s needs and levels of awareness about the topic. In addition, when developing any of the communication tools, they should be evaluated before the complete and continuous implementation to reduce confusion among the public.^8,9^ Especially this type of communication is new to them and acceptance of the information relies on trusted authorities that collect, monitor, and present air quality information to their community members.^28^

## Conclusions

Risk communication is one step to reducing the impact of air pollution in the region. It is a collective effort that needs everyone to be involved.^29^ The public can have significant pressure on governments to enforce bans and impose stricter air quality standards, especially with the current poor air quality indices in most countries of the region. Although the first step is to establish an effective air monitoring system, as indicated by some interviewees, addressing the key points of communication in advance along with possible flaws ensures better outcomes and minimum challenges to encounter. The effective air monitoring system serves as a point of referral, through which experts could advise the public about its usefulness, importance of following and considering any guidelines published by this system, and learning how to benefit from it. However, following the footsteps of previous systems applied in other countries, with considering possible obstacles and issues, should assist in reducing unnecessary steps that might retard the progression of the communication process.

### Limitations

There are some limitations in this study that were identified. First, the study included interviewees who were approached through Snowballing method, which might have missed other experts who could have added valuable information. Second, because of the limited time available to conduct interviews in Lebanon, the numbers of interviewees in both countries were not equal. Third, finding the best time to conduct the interviews was a challenge because of the tight schedule of most experts, which in our belief, had affected the opportunity for some interviewees to provide more comprehensive answers due to time constraints. Finally, generalizability cannot be claimed based on the findings of this study alone but should be accompanied by results of similar studies in the region.

## Appendix 1

Interview guide

Specialty of the expert: _______________________

1. In your opinion what are the health risks associated with air pollution?

Listen to the answer and then stimulate the discussion by asking the following 2 questions.

- What conditions/diseases caused by or affected by air pollutants?
- What are the most vulnerable groups affected by air pollutants?
2. In your opinion, what are the main air pollutants that affect health (cause or aggravate the diseases)? What are the special groups/ or patients who are affected by air pollution?
3. Do you think that we need to communicate/disseminate the air pollution-health risks in the region? Which groups should be targeted?
4. What should be the goals of communicating air pollution-health risks?
5. In your opinion, what are the expected challenges of communication on air pollution such as forecasts, information on observed exceedances of alert thresholds, the types of population concerned, possible health effects, recommended behavior and preventive action to reduce pollution and/or exposure to it).
6. What type of information should be transmitted to the public/target groups?
7. What type of information should be transmitted on the health impacts of air pollution?
• What is the target group for this information?
• How we need to transmit these information (vehicles).
8. What type of information should be transmitted on actions for protecting oneself against the adverse effects of pollution?
• What is the target group for this information?
• How we need to transmit these information (vehicle).
9. What information we need to send to your patients when there is episodes of air pollutants including dust? (Specify the information for each category below).
• Information about possible coping behaviors (eg, rethinking regular routes, avoiding exposure of children in certain areas, etc.) Health education concerning air pollution.
• Actions for protecting the general public.
• Information to sensitive groups (children, adults performing outdoor physical activities, people with chronic respiratory diseases and ozone-sensitive individuals during high pollution episodes, especially during ozone episodes).
• General population’s ability to avoid the risks caused by air pollution.
• Information about possible coping behaviors (eg, rethinking regular routes, avoiding exposure of children in certain areas, etc.).
